# Predicting brain amyloid load with digital and blood-based biomarkers

**DOI:** 10.1186/s13195-025-01801-y

**Published:** 2025-07-05

**Authors:** Weineng Chen, Yu Liao, Xinchong Shi, Fengjuan Su, Haifan Kong, Yingying Fang, Yifan Zheng, Jiayi Zhou, Ganqiang Liu, Xianbo Zhou, Xiaoli Yao, Curtis B. Ashford, Feng Li, Long Yang, Michael F. Bergeron, J. Wesson Ashford, Xiangsong Zhang, Zhong Pei

**Affiliations:** 1 Department of Neurology, The First Affiliated Hospital, Guangdong Provincial Key Laboratory of Diagnosis and Treatment of Major Neurological Diseases, National Key Clinical Department and Key Discipline of Neurology, Sun Yat-sen University, Guangzhou, Guangdong 510080 China; 2 Shenzhen Key Laboratory of Systems Medicine in Inflammatory Diseases, School of Medicine, Shenzhen Campus of Sun Yat-sen University, Shenzhen, Guangdong China; 3 Department of Nuclear Medicine, The First Affiliated Hospital, Sun Yat-sen University, Shenzhen, Guangdong 510080 China; 4 Center for Alzheimer’s Research, Washington Institute of Clinical Research, Vienna, VA, USA; AstraNeura, Co., Ltd., Shanghai, China; 5 MemTrax, LLC, Redwood City, CA USA; 6 Kunming Escher Technology Co. Ltd, Kunming, Yunnan China; 7 Center for Clinical Pharmacy, The First Affiliated Hospital of Kunming Medical University, Kunming, Yunnan China; 8 Department of Health Sciences, University of Hartford, West Hartford, CT USA; 9 Department of Psychiatry & Behavioral Sciences, War Related Illness & Injury Study Center, VA Palo Alto Health Care System, Stanford University, Palo Alto, CA USA

**Keywords:** Alzheimer’s disease, MemTrax, MoCA, Blood biomarkers, Aβ, Centiloid

## Abstract

**Background:**

With the recent approval of anti-β-amyloid (Aβ) treatment for Alzheimer’s disease (AD), a demand has emerged for scalable, convenient and accurate estimations of brain Aβ burden for the detection of AD that would enable timely, accurate and reliable diagnosis in one’s primary care physician’s (PCPs) office as called for recently by World Health Organization (WHO).

**Methods:**

MemTrax, a 2-minute online memory test, was selected as the digital biomarker of cognitive impairment, and blood-based biomarkers (BBMs) including Aβ42, Aβ40, P-tau181, GFAP and NfL were used to estimate AD-related metrics in different groups of elderly individuals (*n* = 349) for comparison with Aβ PET scans of brain Aβ burden. The correlations between MemTrax, MoCA, BBMs and brain Aβ burden, expressed in centiloid (CL) values, were analyzed for predicting CL value alone or in combinations using machine-learning (ML).

**Results:**

Both MemTrax and the MoCA were able to differentiate Aβ status similarly. Integration of MemTrax and BBMs using ML, however, significantly improved the AUCs (over the same with MoCA) for differentiating Aβ status. MemTrax and p-Tau181/Aβ42 composite showed the strongest relationship with CL value among other BBMs. Most importantly, regression analyses of MemTrax and p-Tau181/Aβ42 aptly predicted CL values.

**Conclusion:**

The combination of MemTrax and BBMs provides an accurate, convenient, non-invasive, cost-effective and scalable way to estimate Aβ load, which provides an opportunity for mass screening and timely and accurate diagnosis of AD. Our findings could also facilitate more effective AD clinical management in the PCPs office worldwide for more equitable access to current standard of care.

**Supplementary Information:**

The online version contains supplementary material available at 10.1186/s13195-025-01801-y.

## Background

Alzheimer’s disease (AD) is a progressive and irreversible neurodegenerative disease. The accumulation of extracellular amyloid β (Aβ) plaques and intracellular tangles of hyperphosphorylated tau are pathological hallmarks of the disease. Early diagnosis and intervention are particularly beneficial and important for AD [1]. With the ever-increasing global aging population, the incidence of AD is likewise escalating, which underscores the urgency for the development and implementation of an accessible, affordable and reliable large-scale corresponding screen globally [2]. And with the development of valid digital and blood-based testing, large-scale/mass screening is measurably closer to a feasible and practical reality.

The advent of anti-Aβ immunotherapy after the Food and Drug Administration (FDA) of the United States approved Aducanumab in 2021 [3] further justified timely detection and accurate diagnosis of AD. However, the indications for this treatment specify that the patient must have Aβ deposition in the brain, and the amount of deposition needs to reach a certain centiloid (CL) threshold [4]. Cerebrospinal fluid (CSF) Aβ or positron emission tomography / computed tomography (PET/CT) are the FDA approved tests for the estimation of Aβ deposition in the brain, with the CL value of Aβ deposition accordingly calculated from PET/CT. However, high cost and invasiveness limit the accessibility and application of these procedures. Accurate, convenient, reliable, affordable and accessible methods and timely strategies to detect AD at early stages with the ability to estimate Aβ burden as measured by CL value would be a great advantage in clinically managing AD. Further, such methods could broadly enable timely and accurate diagnosis of AD and widely equip primary care physicians (PCPs) to more aptly provide effective care to early-stage AD patients. These practical developments would also alleviate the unsustainable demand on memory clinics and specialists with the important new focus on recognizing early AD [5].

The utility of blood-based biomarkers (BBMs) for AD has made major progress in recent years and has been shown to be effective in the detection of AD-related pathology for identifying individuals who might benefit from anti-Aβ immunotherapy [6–8]. However, at least 1/3 of individuals with Aβ pathology do not have clinical symptoms during their life span [9], and there is substantial heterogeneity of disease progression. Accordingly, cognitive function impairment in conjunction with measures of AD pathology is a viable direction for identifying and diagnosing early AD patients who may be more optimally suited for guiding treatment with available medicines, particularly the newly approved Aβ antibody drugs. Pan et al. has reported that an integrated algorithm combining plasma biomarkers and traditional cognitive assessments accurately predicts brain Aβ pathology [10]. To make early detection of AD feasible, a more sensitive, convenient and scalable digital cognitive test that could be self-administered would be of great value.

MemTrax is a *de novo* novel picture-based online brief cognitive function test based on a continuous recognition paradigm with a variable *N*-back design intended to detect episodic memory changes in early AD. MemTrax has been extensively cross validated with the MoCA [11] and reference norms published for a large Chinese cohort [12] and a French/European population [13]. Since MemTrax does not require healthcare personnel to administer and takes ~ 2-minutes to complete, it has been adopted for mass memory/brain health screening in both US and China [14, 15]. Our earlier study established that the efficiency of MemTrax in differentiating AD-MCI (mild cognitive impairment) and a healthy control group was markedly improved when combined with AD BBMs, suggesting such a combination may have the potential to predict Aβ burden [16].

In this study, we investigated the utilities of the cognitive assessment digital biomarker MemTrax and BBMs for their respective ability alone or in combination to predict Aβ load measured with PET/CT. This contrasting assessment would support their potential utility in partly replacing or reducing unnecessary PET/CT scans and lumbar punctures for CSF testing when selecting suitable participants for anti-Aβ immunotherapy.

## Methods

### Participants

In this study, a total 349 participants were recruited from Guangzhou health aging and dementia cohort from March 2020 to March 2024, including participants with or without cognitive impairment. All participants underwent detailed cognitive assessment including MMSE, MoCA, Clinical Dementia Rating (CDR), Functional Activities Questionnaire (FAQ), MemTrax, blood tests, and neuroimaging including MRI, PET/CT (Florbetapir and Fludeoxyglucose ). Participants were divided into four groups, including cognitive unimpaired with Aβ negative group (CU Aβ-), MCI with Aβ negative group (MCI Aβ-), MCI with Aβ positive group (MCI Aβ+) and an AD dementia group. The inclusion criteria for CU Aβ- were the absence of objective cognitive symptoms and not fulfilling the criteria for MCI or any dementia disorder, as well as a PET/CT indication of Aβ negative. The inclusion criteria for individuals with MCI were based on the clinical criteria by Petersen with an objective cognitive impairment but fail the criteria of any dementia disorder [17]. The inclusion criteria for AD dementia were based on the criteria published by the National Institute on Aging and Alzheimer’s Association (NIA-AA) with definite Aβ pathology [18].

### Study procedure

For all participants, basic information such as age, sex, years of education, smoking, drinking, past medical history and family history was collected by a member of the study team. MoCA and MemTrax tests were conducted as previously reported [16]. Briefly, MemTrax was conducted after the MoCA test. MemTrax percent correct (%, MTx-%C) and mean response time (in seconds, MTx-RT) were recorded for each participant. Two members of the study team verified the completed questionnaire and the results of MoCA separately.

### MemTrax test

Detailed description of the theory and design of MemTrax has been published previously [13]. Briefly, with each MemTrax test, participants were asked to identify repeated images among a series of 50 images (25 new images and 25 repeated images). Each image was shown for three seconds or until participants responded and touched the screen when repeated images were shown. At the end of the test, the program calculated and showed the MTx-%C and the MTx-RT of all correct responses. The MemTrax composite score (MTx-Cp) was derived by multiplying the numbers in MTx-%C and the reciprocal of MTx-RT. MemTrax was administered on a computer using the web-portal located on a cloud server in China (http://www.memtrax.com.cn). MemTrax is also available at www.memtrax.org.

### Plasma collection and assays

Blood samples were collected after overnight fasting from all participants at each respective initial visit. After centrifugation at 4000 rpm, 10 min (4 °C), each plasma sample was collected and stored at -80℃. BBMs: Aβ40, Aβ42, p-Tau181, GFAP and NfL, were later quantified using an ultra-sensitive Simoa technology (Quanterix, MA, US) on the automated Simoa HD-X platform (GBIO, Hangzhou, China) according to the manufacturer’s instruction. The Neurology 4-Plex E Assay Kit (Cat No:103670), p-Tau181 Advantage V2 assay kit (Cat No:103714) were purchased from Quanterix and used as directed. Plasma samples were diluted with sample diluent included in the kits at a 1:4 ratio for measurement. Calibrators and quality controls were measured in duplicate. All sample assays and measurements were performed on a single run using kits from the same lot number. Operators were blinded to participants’ disease status.

### Amyloid PET and structural brain MRI

Participants underwent PET/CT and MRI scans at the First Affiliated Hospital of Sun Yat-sen University. The Aβ imaging agent used for assessing Aβ deposition was 18 F-Florbetapir, synthesized in the Radiopharmaceutical Center of the Department of Nuclear Medicine at this hospital, following a previously established protocol [19]. The synthesis was performed using an ALLINONE automated synthesis module. The final product was quality-controlled using HPLC before being used for imaging. PET scans were acquired using a GE Discovery MI scanner. Each participant received an intravenous injection of 10 mCi of 18 F-Florbetapir, followed by a 60-minute rest period before PET acquisition. The scan began with a low-dose CT scan for attenuation correction, followed by a 10-minute PET acquisition. Final PET images were reconstructed using a super-iterative reconstruction algorithm. Each participant also underwent a 3D T1-weighted magnetic resonance imaging (MRI) scan using a SIEMENS MAGNETOM Prisma (3T) scanner for assessing cortical volume in different brain regions and a corresponding global brain atrophy index. MRI scans were conducted within 1 month after each respective PET scan.

### CL and definition of Aβ status

For each participant, CL values were calculated to semi-quantitatively assess Aβ deposition in the brain. The process of calculating CL values was based on a previous study [20]. Briefly, the PET image of each participant was first registered to the corresponding MRI T1 image. The T1 image was then normalized to the Montreal Neurological Institute (MNI)-152 space using Statistical Parametric Mapping version 12 (SPM12). The transformation parameters were then applied to the registered PET image, normalizing it to the MNI-152 coordinate system. Finally, regions of interest (ROIs) were extracted from the cerebral cortex and the whole cerebellum. Average counts were calculated for each ROI, and the cortical-to-cerebellar ratio (SUVr) was calculated and converted to a CL value. Previous studies have established a threshold of CL > 20 as indicative of Aβ positivity [21–23]. Therefore, this study also used 20 as the cut-off for determining Aβ positivity in these participants.

### Statistical analyses

Statistical analyses were conducted using R version 4.2.2 and MATLAB. All figures were also generated in R. Data preprocessing included outlier removal to ensure robustness. Outliers were defined as values beyond the first quartile (Q1) minus 3.0 times the interquartile range (IQR) or the third quartile (Q3) plus 3.0 × IQR for blood markers, and were excluded from subsequent analyses. For comparing variables between two groups, baseline demographic and clinical data, along with biomarker levels, were analyzed using the Chi-square test for categorical variables and the Mann-Whitney U test for continuous variables. For comparisons involving multiple groups, *P*-values for continuous and categorical variables were determined using analysis of variance (ANOVA) and the Chi-square test, respectively. All *P*-values were two-tailed, with statistical significance established at *P* < 0.05.

The correlations between MemTrax, MoCA, and levels of plasma biomarkers were assessed using Pearson correlation coefficients. Logistic regression was employed for group predictions using the glm function with a binomial logit link. This approach corresponds to standard (unregularized) logistic regression. The dataset was relatively balanced in terms of Aβ status, with 171 Aβ + individuals (comprising 85 MCI Aβ + and 86 AD dementia participants) and 178 Aβ- individuals (comprising 91CU Aβ- and 87 MCI Aβ- participants). Given this balance, no additional techniques for handling class imbalance were deemed necessary. Additionally, receiver-operating characteristic (ROC) analyses, along with the estimation of the area under the curve (AUC) and 95% confidence intervals (CIs), were performed using the “pROC” package [24]. Furthermore, we employed logistic regression to estimate each subject’s probability of amyloid PET positivity, using digital and blood biomarkers. Logistic regression models were also performed five-fold cross validation algorithm. Specifically, the data were randomly divided into five equal segments: four for training and one for testing. This process was repeated ten times utilizing a different segment as the test set (Supplementary Tables 1, 2).

*P*-value from DeLong’s test comparing the AUC of the biomarker was used to assess significant differences in predictive performance. We derived individualized Aβ + probabilities (range: 0–1) via logistic regression. Risk stratification was achieved by defining high and low probability cut-off, optimized to meet pre-specified clinical performance criteria: sensitivity ≥ 90%, specificity ≥ 75%, and an intermediate-risk subgroup < 20% of the cohort. Subjects were classified as high-risk (probability > high cut-off), low-risk (probability < low cut-off), or intermediate-risk (probability between cut-offs) [25].

The sensitivity, specificity and AUC using the “psych” package were calculated to evaluate the performance of continuous digital and plasma biomarker levels in classifying Aβ positivity among subjects (Aβ- vs. Aβ+). MATLAB-based algorithms were used for feature ranking to assess the absolute contribution of each variable. The ‘fsrftest’ function in MATLAB was specifically applied to predict continuous variables such as plasma biomarker levels.

MemTrax, p-Tau181, GFAP, and other biomarkers were used to predict CL values using linear regression methods. Logarithmic transformations were applied to all plasma biomarkers to better approximate a normal distribution.

## Results

### Demographic and descriptive statistics

The study cohort consisted of 349 elderly participants, of which 171 were Aβ positive on PET/CT and 178 were Aβ negative. Baseline demographic and descriptive statistics are reported in Table 1. The two groups were similar in sex distribution (*p* = 0.1979) while the Aβ negative group was older (*p* = 0.0410). Years of education was higher in the Aβ- group (*p* = 0.0018). In this cohort, smoking (*p* = 0.0243) was more common in Aβ + group, but not alcohol use (*p* = 0.5433). In addition, more Aβ- participants had past histories of hyperlipidemia (*p* = 0.0261), while more Aβ + participants had family histories of cognitive deficit (*p* = 0.0340). No difference in medical history of hypertension (*p* = 0.2197) or diabetes (*p* = 0.4506) were found between the groups.

**Table 1:** Baseline demographic and clinical characteristics and biomarker values

	Aβ+(*n* = 171)	Aβ−(*n* = 178)	*p*-Value
Age, years M(SD)	63.43(8.858)	65.39(6.984)	**0.0410**
Female, n (%)	98(57.31)	115(64.61)	0.1979
Education, years M(SD)	9.985(4.022)	11.04(3.745)	**0.0018**
Smoking, n (%)	40(23.39)	24(13.48)	**0.0243**
Alcohol, n (%)	28(16.37)	24(13.48)	0.5433
Hypertension, n (%)	40(23.39)	53(29.78)	0.2197
Diabetes, n (%)	22(12.87)	29(16.29)	0.4506
Hyperlipidemia, n (%)	10(5.85)	24(13.48)	**0.0261**
Family history of AD, n (%)	60(35.09)	43(24.16)	**0.0340**
CDR, M(SD)	0.8099(0.3840)	0.2444(0.2506)	**<0.0001**
MMSE, M(SD)	20.15(7.064)	27.74(3.508)	**<0.0001**
MoCA, M(SD)	14.50(7.227)	23.01(5.315)	**<0.0001**
MTx-%C	67.59(11.15)	80.02(9.18)	**<0.0001**
MTx-RT	1.609(0.3533)	1.405(0.2413)	**<0.0001**
MTx-Cp	45.23(18.92)	58.81(13.39)	**<0.0001**
Aβ42/40, M (SD)	0.05606(0.0122)	0.07089(0.01355)	**<0.0001**
P-Tau181(pg /ml), M (SD)	3.777(1.651)	1.882(0.903)	**<0.0001**
NfL (pg /ml), M (SD)	22.95(9.81)	16.87(8.59)	**<0.0001**
GFAP (pg/ml), M (SD)	153.74(70.94)	83.97(48.12)	**<0.0001**

Data for MoCA are presented for 172Aβ−, 157Aβ + individuals, Data for MemTrax are presented for 173Aβ−, 131Aβ + individuals, Data for blood biomarkers are presented for 161Aβ−, 127Aβ + individuals

As shown in Table 1, traditional and digital cognitive tests including CDR, MMSE, MoCA, MTx-%C, MTx-RT, MTx-Cp were all significantly better in Aβ − group. Among the three indexes of digital test, MTx-%C (AUC = 0.802, 95% CI: 0.753–0.851) is better compared with MTx-RT (AUC = 0.688, 95% CI: 0.627–0.750, *p* = 0.0005), while not significantly better than MTx-Cp (AUC = 0.769, 95% CI: 0.714–0.824, *p* = 0.134) for differentiating AD using DeLong’s test. Comparison of AD-related blood biomarkers showed that Aβ42/40 is remarkably lower in Aβ + group. The concentration of p-Tau181, GFAP and NfL were significantly higher in Aβ + group compared with Aβ- group.

### Modeling digital and blood biomarkers in differentiating Aβ status

**Fig. 1:**
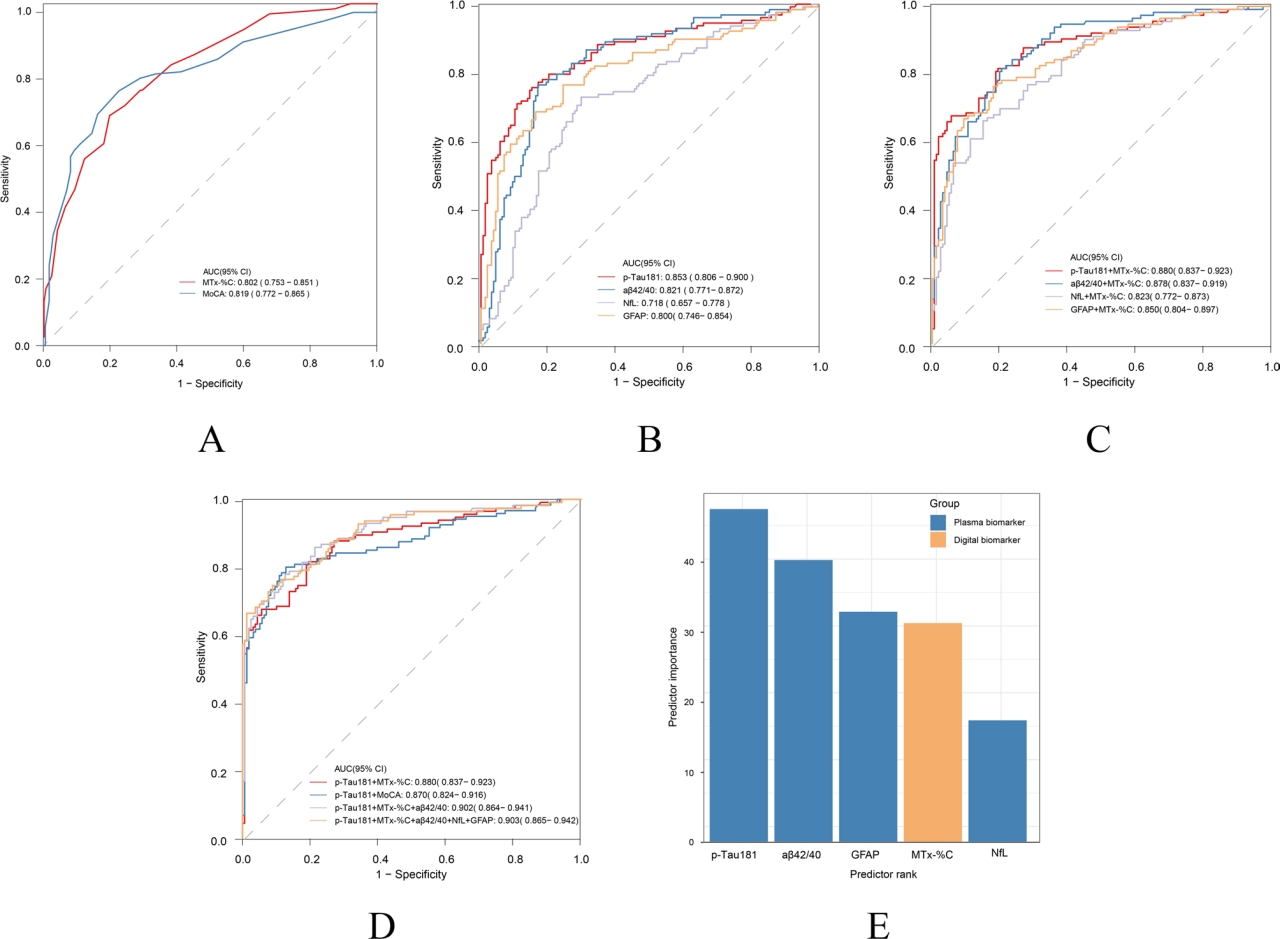
MemTrax and blood-based biomarkers (BBMs) in differentiating Aβ status. (**A**) Receiver-operating characteristic (ROC) curves for distinguishing Aβ status by MemTrax (MTx-%C) and MoCA. (**B**) ROC curves for differentiating Aβ status by p-Tau181, Aβ42/40, NfL and GFAP. (**C**) ROC curves for differentiating Aβ status by combining MemTrax and blood biomarkers. (**D**) ROC curves comparison of MemTrax and MoCA when adding blood biomarkers. (**E**) Ranked BBMs and digital cognitive metrics in predicting Aβ + or Aβ-. BBMs are marked in orange, while online cognitive metrics are marked in blue

**Fig. 2:**
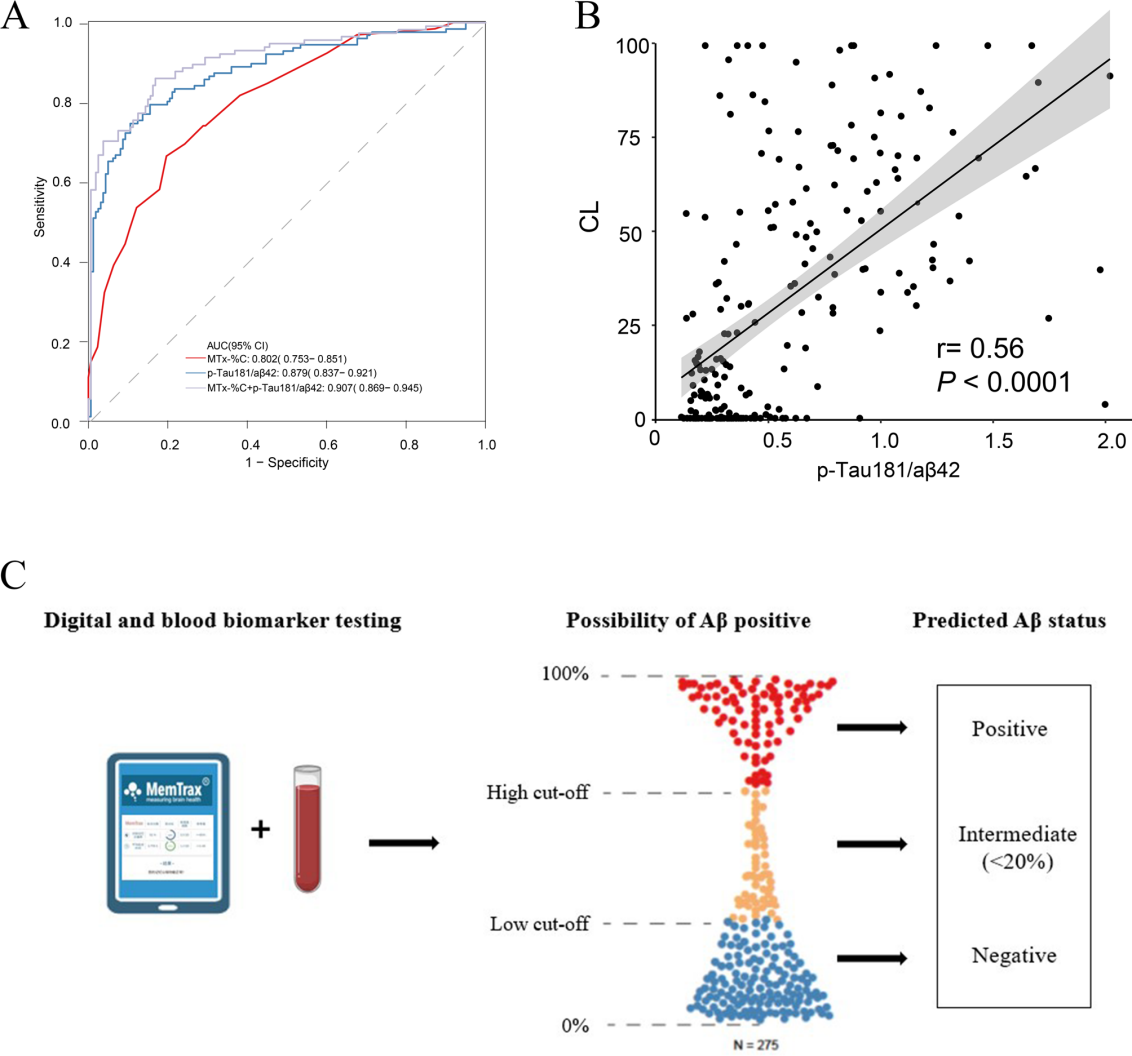
The two cut-off approach for testing combing digital and blood-based biomarker of Aβ pathology. (**A**) Combining MemTrax (MTx-%C) and p-Tau181/Aβ42 in differentiating Aβ status. (**B**) The correlation between p-Tau181/Aβ42 and CL value. (**C**) Usage of a two-cut-off approach for testing combining MemTrax and p-Tau181/Aβ42 leads to three categories of results: positive, intermediate and negative, increasing the accuracy with which people can be classified as Aβ+/- in PET-CT scan. Individuals classified as having intermediate results are no more than 20%

The capability of MemTrax in differentiating Aβ status is shown in Fig. 1. The AUC of MTx-%C was 0.802 (95% CI: 0.753–0.851), which showed no statistically significant difference compared to that with MoCA (AUC = 0.819, 95% CI: 0.772–0.865) as assessed by DeLong’s test (*p* = 0.726). Multivariable logistic analyses indicated that MemTrax significantly enhanced the AUC of BBMs. The AUC of the best blood biomarker p-Tau181 was 0.853(95% CI: 0.806–0.900), with a sensitivity of 0.754, specificity of 0.844, positive predictive value (PPV) of 0.791, and negative predictive value (NPV) of 0.813. When combined with MTx-%C, the AUC increased to 0.880 (95% CI: 0.837–0.923, DeLong’s test for AUC comparison, *p* = 0.03), accompanied by improved sensitivity (0.816), NPV (0.861) and decreased specificity (0.805) and PPV (0.750) (Supplementary Table 3). In contrast, the enhancing effect of MoCA on Tau181 (from 0. 853 to 0.870, DeLong’s test for AUC comparison, *p* = 0.14) was not significant. Moreover, when incorporating MemTrax, the AUC of other blood biomarkers including Aβ42/40 (*p* = 0.006), GFAP (*p* = 0.003), NFL (*p* = 0.0001) and p-Tau181/ Aβ42 (*p* = 0.018) also had varying degrees of improvement (Figs. 1 and 2). When grading predictor importance in differentiating Aβ status, we found p-Tau181 to have the highest, followed by Aβ42/40, GFAP, MTx-%C and NfL (Fig. 1E).

### Correlations between cognitive tests, blood-based biomarkers and CL value

**Fig. 3:**
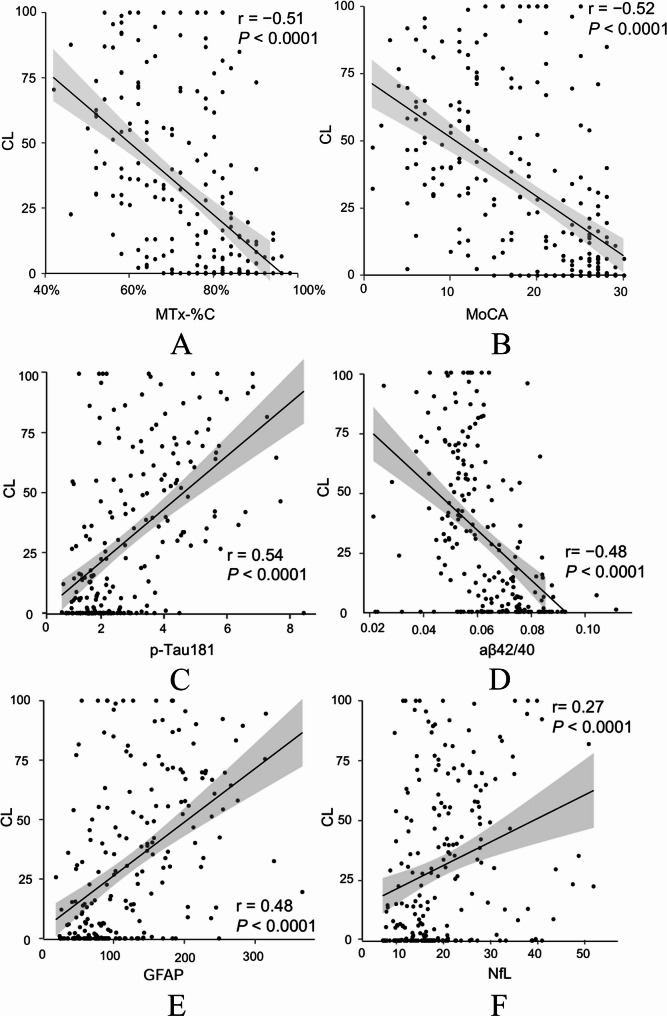
Pearson correlation between CL and (**A**) MemTrax (MTx-%C). (**B**) MoCA, (**C**) p-Tau181, (**D**) Aβ42/40, (**E**) GFAP, (**F**) NfL. Aβ, amyloid β; GFAP, glial fibrillary acidic protein; NfL, neurofilament light

To determine variables to predict a concrete CL value, the correlations between digital and conventional cognitive tests, blood biomarkers and the CL values were analyzed. All markers were found to be individually correlated with CL values (Fig. 3). MTx-%C and MoCA demonstrated similar higher correlations in comparison to BBMs and both were negatively correlated with CL values (*r *= -0.51, *p*<0.0001 and *r *= -0.52, *p*<0.0001, respectively). Among the AD-related BBMs, p-Tau181 exhibited the strongest correlation with CL values (*r* = 0.54, *p*<0.0001), followed by GFAP (*r* = 0.48, *p*<0.0001), Aβ42/40 (*r *= -0.48, *p*<0.0001) and NfL (*r* = 0.27, *p*<0.0001).

### MemTrax and BBMs in differentiating Aβ status between different groups

All participants were divided into CU Aβ-, MCI Aβ-, MCI Aβ + and AD dementia groups based on respective cognitive function and Aβ status. Basic characteristics are presented in Table 2. Briefly, education years, cognitive tests and blood biomarkers were significantly different among different groups.

**Table 2:** Participant characteristics among different subgroups

	CU Aβ-(91)	MCI Aβ-(87)	MCI Aβ+(85)	AD dementia(86)	*p*-Value
Age, years M(SD)	65.97(4.891)	64.78(8.638)	65.78(7.587)	61.12(9.441)	**<0.0001**
Female, n (%)	67(73.63)	48(55.17)	46(54.12)	52(60.47)	**0.0287**
Education, years M(SD)	11.89(2.909)	10.15(4.296)	11.56(3.994)	8.465(3.439)	**<0.0001**
CDR, M(SD)	0(0.000)	0.5(0.000)	0.5(0.000)	1.116(0.3224)	**<0.0001**
MMSE, M(SD)	29.35(0.7207)	25.98(4.401)	24.99(4.395)	15.08(5.629)	**<0.0001**
MoCA, M(SD)	25.77(2.785)	19.91(5.769)	18.87(6.231)	9.333(4.344)	**<0.0001**
MTx-%C	83.64(7.310)	76.00(9.397)	72.98(9.424)	58.57(7.360)	**<0.0001**
MTx-RT	1.388(0.2327)	1.423(0.2507)	1.552(0.3389)	1.705(0.3596)	**<0.0001**
MTx-Cp	62.07(12.40)	55.16(13.59)	49.60(12.45)	37.94(24.02)	**<0.0001**
Aβ42/40, M (SD)	0.07278(0.01547)	0.06873(0.01065)	0.05708(0.01323)	0.05479(0.01076)	**<0.0001**
P-Tau181 (pg/ml), M (SD)	1.804(0.670)	1.970(1.108)	3.272(1.519)	4.408(1.604)	**<0.0001**
NfL (pg /ml), M (SD)	15.62(7.04)	18.34(9.97)	21.09(10.29)	25.35(8.65)	**<0.0001**
GFAP (pg/ml), M (SD)	79.93(43.51)	88.60(52.83)	128.0(60.87)	185.94(69.92)	**<0.0001**

Data for MoCA are presented for 91CU Aβ-, 81MCI Aβ-, 85MCI Aβ+, and 72 AD dementia individuals, Data for MemTrax are presented for 91CUAβ-, 82MCI Aβ-, 82MCIAβ+, and 49 AD dementia individuals, Data for blood biomarkers are presented for 86CUAβ-, 75MCIAβ-, 70MCIAβ+, and 57 AD dementia individuals

**Fig. 4:**
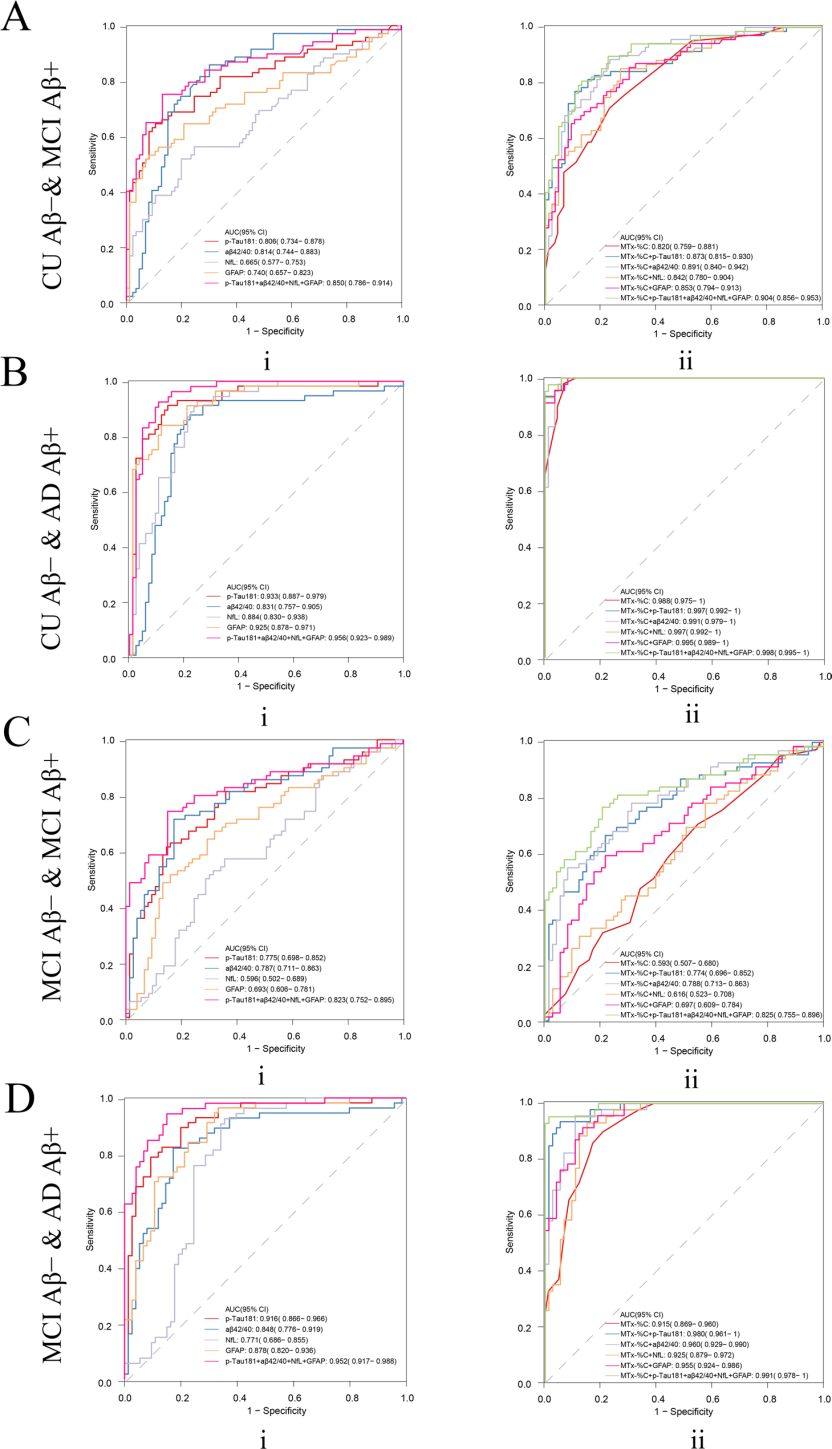
Receiver-operating characteristic (ROC) curves for distinguishing between (**A**) CU Aβ- and MCI Aβ+, (**B**) CU Aβ- and AD, (**C**) MCI Aβ- and MCI Aβ+, and (**D**) MCI Aβ- and AD dementia participants. ROC curves are presented for A, B, C, and D for (i) Aβ42/ 40, p-Tau181, GFAP, NfL and Aβ42/ 40 + p-Tau181 + GFAP + NfL and (ii) model comprising MemTrax (MTx-%C) + Aβ42/40, MemTrax + p-Tau181, MemTrax + GFAP, MemTrax + NfL, MemTrax + Aβ42/40 + p-Tau181 + GFAP, and MemTrax + Aβ42/40 + p-Tau181 + GFAP + NfL

To clarify the role of different blood markers and MemTrax in different stages of the AD continuum and to find the simplest and best model in predicting Aβ status, we performed more detailed groupings and conducted corresponding analyses. Here the performance among different groups by different blood biomarkers was explored. Combining Aβ42/40 and MTx-%C was found to be best for differentiating CU Aβ- and MCI Aβ+, with an AUC of 0.891(95% CI: 0.840–0.942), a sensitivity of 0.884, specificity of 0.767, PPV of 0.753, and NPV of 0.892. Furthermore, combination of p-Tau181 and MTx-%C performed best to predict their brain amyloid load on the rest of the subgroup comparisons including CU Aβ- and AD dementia (AUC = 0.997, 95% CI: 0.992–1), with sensitivity of 0.999, specificity of 0.942, PPV of 0.902, and NPV of 0.999, as well as MCI Aβ- and AD dementia (AUC = 0.980, 95% CI: 0.961–1), with sensitivity of 0.935, specificity of 0.946, PPV of 0.915, and NPV of 0.959 (Fig. 4, Supplementary Table 3). However, when differentiating MCI Aβ- and MCI Aβ+, neither BBMs nor MemTrax showed good efficacy (Fig. 4C).

### Two cut-off approach for multiple-model test of Aβ status

CSF testing is regarded as a benchmark for Aβ pathology for BBM test performance and is approved by FDA [26]. To match this criterion, a two-cut-off approach for blood test has been reported to classify patients into three categories of results: positive, intermediate and negative [25]. As previous study reported p-Tau181/ Aβ42 performed best in predict Aβ-PET status among other biomarkers including Aβ42/40 and p-Tau181 [27]. For clinical application, we first verified p-Tau181/ Aβ42’s performance in this cohort (Fig. 2). The AUC in differentiating Aβ status (AUC = 0.879) and the correlation with CL value (*r* = 0.56) outperformed single BBMs. Here, we found a multiple-model combining MTx-%C and p-Tau181/ Aβ42 applies well to the two-cut-off approach in differentiating Aβ status, which makes it more accessible for clinical usage (Fig. 2). According to Supplementary Table 4, the cut-off included meeting the criteria of a sensitivity of ≥ 90% with a specificity of ≥ 75%. The best cut-off value is 0.17–0.43 or 0.44, with the highest sensitivity of 90.7% and specificity of 84.1% with a PPV of 0.815 and a NPV of 0.922. With this approach, no more than 18.55% of individuals would be classified as having intermediate results requiring further testing. For clinical convenience, we formed a heat map showing the predicted Aβ status by MTx-%C and p-Tau181/ Aβ42 (Supplementary Fig. 1).

### Multifactorial modeling in predicting Aβ burden

**Fig. 5:**
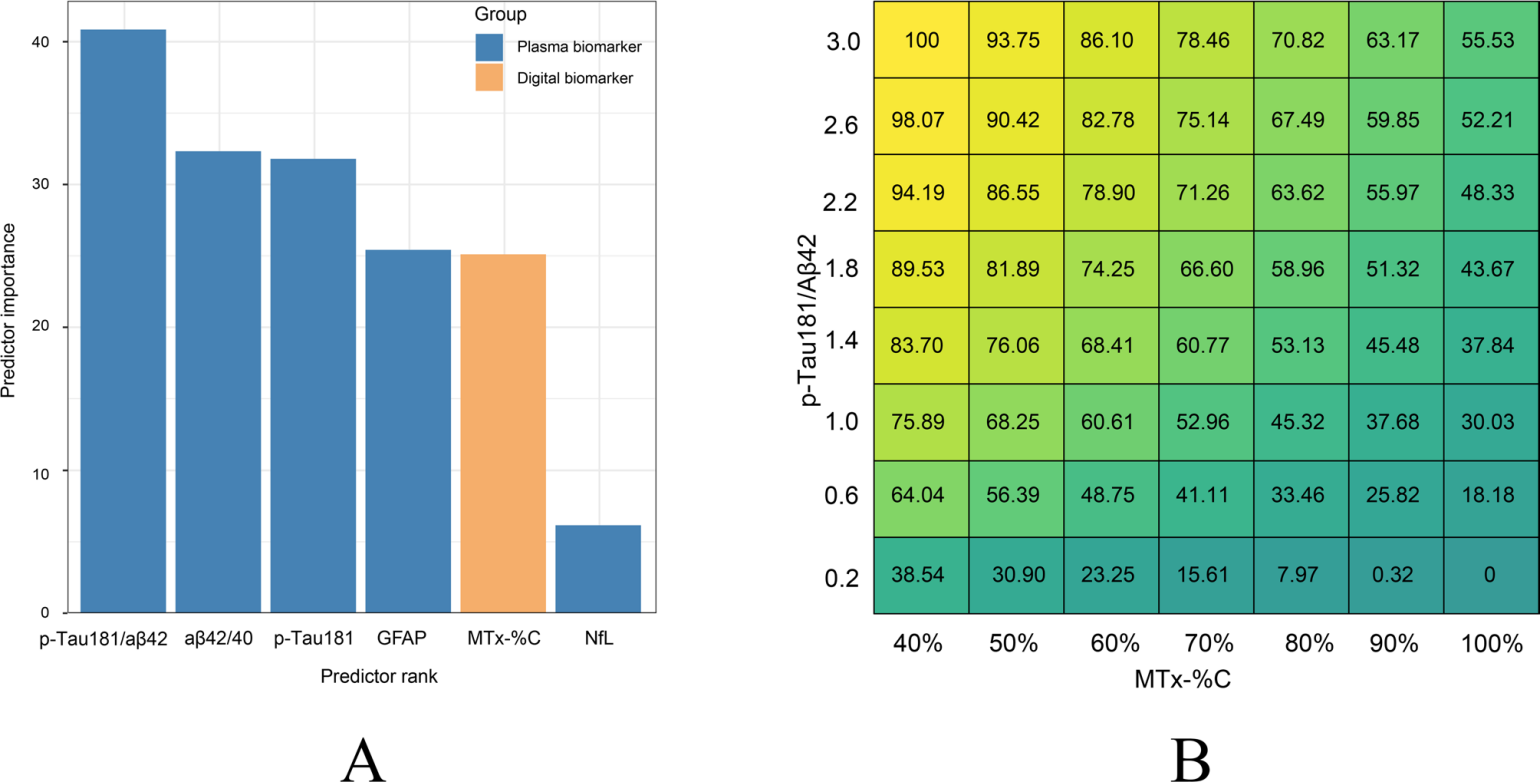
Digital and BBMs in predicting CL value. (**A**) Ranked biomarkers and digital cognitive metrics in predicting CL value. Plasma biomarkers are marked in orange, while online cognitive metrics are marked in blue. (**B**) Heat map showing predicted CL value by combining p-Tau181/Aβ42 and MemTrax (MTx-%C) using linear regression

CL values are important for selecting patients for anti-Aβ treatment. The selected variables were then ranked according to their importance in predicting a CL value. In our cohort, the p-Tau181/ Aβ42 ratio is the best predictor among blood biomarkers (Fig. 5A). Multifactorial modeling with regression analyses combining MTx-%C and BBMs were performed to determine their suitability to predict or estimate CL value. Among the five linear regression models in predicting CL value, the combination of MTx-%C and p-Tau181/ Aβ42 was the best model with the highest Coefficient of Determination (R^2^ = 0.436) (Table 3). For clinical convenience and utilities, we formed a heat map showing the predicted CL value by MTx-%C in combination with p-Tau181/ Aβ42 (Fig. 5B).

**Table 3:** Prediction of CL value by negative binomial distribution generalized linear regression fitting

*N* = 205(NfL = 204)	Model	*p*-value					
	R^2^	MemTrax	p-Tau181	NfL	GFAP	Aβ42/40	p-Tau181/ Aβ42
Model1	0.359	***p*** **< 0.001**	***p*** **< 0.001**				
Model2	0.349	***p*** **< 0.001**				***p*** **< 0.001**	
Model3	0.323	***p*** **< 0.001**			***p*** **< 0.001**		
Model4	0.265	***p*** **< 0.001**		**0.013**			
Model5(best model)	0.436	***p*** **< 0.001**					***p*** **< 0.001**

## Conclusion

We demonstrated that a 2-minute digital cognitive assessment combined with BBMs successfully estimates Aβ status and accurately predicts amyloid load in early AD patients. These findings extend our earlier results that similar composites identified clinically diagnosed MCI-AD with AUC up to 0.975 [16]. A recent proposed change in the definition of AD [28] includes AD biology/pathology without any clinical symptoms at the early stages of the AD continuum. Coincident with the greater availability of Aβ immunotherapy that is most effective for early AD, it is pivotal that early AD patients can be identified conveniently, reliably and accurately in a timely way with affordable and accessible means preferably standardized for their adoption globally. To achieve this vision, the advent of digital cognitive assessments and BBMs are the most promising new technologies. While BBMs remain less accurate than PET-CT, and CSF analyses have limited clinical use, significant progress has been made. Digital biomarkers show promise but face challenges in standardization and adaptability. Their accuracy approaches, but does not exceed, traditional cognitive scales. We and others have shown that combining BBMs with traditional and digital cognitive assessments, respectively could correctly classify AD [10]. A recent study further showed that models combing a different set of digital tests and BBMs are capable to discriminating Aβ status [29]. Besides, more and more digital biomarkers such as the Boston Remote Assessment for Neurocognitive Health (BRANCH) and the Face-Name Associative Memory Exam (FNAME) have also been reported to be associated with Aβ burden, which also increases the feasibility of combining blood with digital biomarkers to predict Aβ burden [30, 31].

MemTrax is a rapid and convenient digital cognitive screening test which our previous study found to be efficient in differentiating healthy controls and MCI-AD alone or in combination with BBMs [16]. In this study, the ability of MemTrax to identify Aβ pathology and estimate its burden was explored. MemTrax was found not only as good as MoCA in differentiating Aβ status, but also significantly enhance the AUC of BBMs in differentiating Aβ status. Although the increase in AUC with the addition of MemTrax is numerically small, it nevertheless represents significant value in confirming an AD patient with both disease biology and cognitive function impairment, i.e., a patient with early symptoms and AD pathology. Given that MemTrax can be self-administered in 2-minutes with automatic scoring and results storage, using images as stimuli to minimize the influence of culture and language for uniform adaptation globally, it has the potential to be suitable for AD mass screening globally that could greatly enhance AD early detection and clinical management.

In this study, the participants were further subdivided into different cognitive impairment groups along the AD continuum for identification of Aβ status. MemTrax was found to be effective in differentiating AD dementia or MCI Aβ + from CU Aβ- and AD dementia from MCI Aβ-. Further, MemTrax significantly enhanced the performance of blood biomarkers in differentiating Aβ status between the subgroups. However, the performance of MemTrax in identifying Aβ status between MCI Aβ + and MCI Aβ-was inadequate for clinical use. This likely occurs because MCI diagnosis relies on cognitive test results rather than Aβ status, whereas MemTrax specifically evaluates cognitive performance, not Aβ levels.

To ensure its effectiveness in clinical applications, we tested MemTrax performance and combination of MemTrax with p-Tau181/ Aβ42, to determine which (if any) tests could meet the acceptable performance criteria proposed by The Global CEO Initiative (CEOi) on Alzheimer’s Disease BBM Working group in screening tests. In our cohort, the optimal lower cutoff was 0.17, while the optimal higher cutoff was 0.43 or 0.44, with the highest sensitivity of 90.8% and specificity of 84.1% with a positive predictive value (PPV) of 0.817 and a negative predictive value (NPV) of 0.922. With this approach, less than 18.5% of individuals would be classified as having intermediate results requiring further testing for potential DMT treatment.

With the availability of anti-Aβ immunotherapy, Aβ burden has become a central issue for selecting suitable patients for treatment and for follow up assessments. Traditionally, PET/CT scan has been used to calculate CL values for the inclusion of participants for anti-Aβ treatment in clinical trials, with a precise threshold being required to define Aβ positivity [4, 32]. As reported in our study here, a strong association between MemTrax and CL values was found, which makes it possible to predict Aβ burden when combining BBMs. We also found that the contribution of p-Tau181/ Aβ42 for the estimation of CL value was the highest among BBMs. Moreover, its combination with MemTrax for the estimation of Aβ burden demonstrated the greatest potential among BBMs tested with a heat map generated to guide clinical utilities.

The key contributions of this study lie in five factors. First, the digital biomarker MemTrax was correlated with CL values, and MemTrax was found to be effective in identifying true Aβ negative PET/CT scans. Second, the accuracy of Aβ status determination of BBMs can be improved by incorporating MemTrax, which is better than the results obtained with another digital test reported recently [29]. This combination could facilitate large-scale AD screening targeting middle-aged and elderly people. Thirdly, the brain Aβ burden can be estimated through the combination of MemTrax and BBMs, which cannot only exclude patients who are likely Aβ negative but also facilitate screening for patients with CL > 30 for clinical trials of anti-Aβ immunotherapy and for clinical management of AD with Aβ antibody therapy. The potential reduction of needs for PET/CT and CSF tests with the adaptation of MemTrax in combination with BBMs such as p-Tau181/Aβ42 tests as reported here offers a less expensive and more accessible means for the needs of identifying AD patients with AD biology and early symptoms indicated for the treatment with the newly approved amyloid antibody therapies. Fourthly, our findings here may facilitate earlier and reliable diagnosis of AD in PCPs’s office. Lastly, the participants in our study are all Chinese speaking Chinese ethnicity which provides diversity to the literature dominated with studies from English speaking European ethnicities, though future studies with other racial ethnicities and languages are desired to solidify our findings.

Several caveats pertain to our findings. Firstly, the healthy control group has a relatively high proportion of women and high levels of education, which may introduce bias. Subsequent studies with a larger and more sex balanced cohort could mitigate the influence of this imbalance. Secondly, this is a cross-sectional design, which precludes assessment of individual cognitive trajectories. Longitudinal studies with serial assessments are required to validate these findings and establish their predictive utility. Thirdly, our study population was predominantly Chinese, which limits the generalizability of our results to other ethnic groups. Given established ethnic variations in AD biomarkers, validation in diverse ethnic cohorts is imperative prior to clinical implementation, which would simultaneously address generalizability and overfitting concerns.

In conclusion, our findings indicate that MemTrax has the capability to identify AD patients and Aβ status, alone and with improved AUC when administered in combination with BBMs. Further, the combination could identify early AD patients with both AD biology and cognitive function impairment. And the combination of MemTrax and BBMs is optimal for CL value prediction which could aid in the selection of suitable patients for anti-Aβ immunotherapy and clinical management of AD. Most importantly, our findings provide an urgently needed solution for a simple, convenient, affordable and accessible timely way to accurately detect and diagnose AD in PCPs globally.

## Electronic supplementary material

Below is the link to the electronic supplementary material.

Supplementary Material 1

## Data Availability

No datasets were generated or analysed during the current study.

## Abbreviations

Aβ: β-amyloid
AD: Alzheimer’s disease
AUC: area under the curve
BBMs: blood-based biomarkers
CDR: Clinical Dementia Rating
CL: centiloid
CSF: cerebrospinal fluid
FAQ: Functional Activities Questionnaire
MCI: mild cognitive impairment
MTx-%C: MemTrax percent correct
MTx-RT: MemTrax mean response time
MTx-Cp: MemTrax composite score
MRI: magnetic resonance imaging
NPV: negative predictive value
PCPs: primary care physicians
PET/CT: positron emission tomography / computed tomography
PPV: positive predictive value
ROC: receiver-operating characteristic
WHO: World Health Organization
