# Supplementary material for: Predicting brain amyloid load with digital and blood-based biomarkers
Source: Alzheimers Res Ther. 2025 Jul 5;17:149. doi: 10.1186/s13195-025-01801-y (PMC12228144; doi:10.1186/s13195-025-01801-y)
Supplement: Supplementary file 1 — Supplementary Material 1 [file 13195_2025_1801_MOESM1_ESM.docx]

**Supplementary materials:**

**Table 1**: AUC values of five-fold cross-validation to classify Aβ+ and Aβ-

| Variable(s) | AUC | 95%CI low | 95%CI upper |
| --- | --- | --- | --- |
| MTx-%C | 0.802 | 0.788 | 0.816 |
| MoCA | 0.821 | 0.807 | 0.835 |
| p-Tau181 | 0.852 | 0.838 | 0.866 |
| aβ42/40 | 0.821 | 0.806 | 0.836 |
| NfL | 0.717 | 0.699 | 0.734 |
| GFAP | 0.800 | 0.784 | 0.816 |
| p-Tau181+ MTx-%C | 0.877 | 0.864 | 0.890 |
| aβ42/40+ MTx-%C | 0.874 | 0.860 | 0.887 |
| NfL+ MTx-%C | 0.819 | 0.805 | 0.834 |
| GFAP+ MTx-%C | 0.847 | 0.834 | 0.861 |
| p-Tau181+MoCA | 0.868 | 0.853 | 0.883 |
| p-Tau181+ MTx-%C + aβ42/40 | 0.896 | 0.884 | 0.908 |
| p-Tau181+ MTx-%C + aβ42/40+NfL+GFAP | 0.896 | 0.883 | 0.908 |
| p-Tau181 | 0.807 | 0.783 | 0.830 |
| aβ42/40 | 0.813 | 0.791 | 0.835 |
| NfL | 0.660 | 0.632 | 0.689 |
| GFAP | 0.739 | 0.717 | 0.762 |
| p-Tau181 + aβ42/40+NfL+GFAP | 0.830 | 0.807 | 0.853 |
| p-Tau181+ MTx-%C | 0.869 | 0.850 | 0.888 |
| aβ42/40+ MTx-%C | 0.884 | 0.870 | 0.899 |
| NfL+ MTx-%C | 0.840 | 0.825 | 0.856 |
| GFAP+ MTx-%C | 0.851 | 0.833 | 0.870 |
| p-Tau181+ MTx-%C + aβ42/40+NfL+GFAP | 0.888 | 0.872 | 0.905 |
| p-Tau181 / aβ42 | 0.880 | 0.868 | 0.891 |
| p-Tau181 / aβ42+ MTx-%C | 0.903 | 0.892 | 0.915 |

**Table 2**: AUC values of five-fold cross-validation to classify Aβ+ and Aβ- in different group

|  | CU- VS MCI+ | | | CU- VS AD | | | MCI- VS MCI+ | | | MCI- VS AD | | |
| --- | --- | --- | --- | --- | --- | --- | --- | --- | --- | --- | --- | --- |
| Variable(s) | n = 176 | | | n = 177 | | | n = 172 | | | n = 173 | | |
|  | AUC | 95%CI  low | 95%CI  upper | AUC | 95%CI  low | 95%CI  upper | AUC | 95%CI  low | 95%CI  upper | AUC | 95%CI  low | 95%CI  upper |
| p-Tau181 | 0.807 | 0.783 | 0.830 | 0.932 | 0.916 | 0.948 | 0.771 | 0.751 | 0.791 | 0.915 | 0.900 | 0.931 |
| aβ42/40 | 0.813 | 0.791 | 0.835 | 0.833 | 0.810 | 0.855 | 0.789 | 0.767 | 0.810 | 0.850 | 0.828 | 0.873 |
| NfL | 0.660 | 0.632 | 0.689 | 0.885 | 0.872 | 0.898 | 0.623 | 0.595 | 0.651 | 0.776 | 0.750 | 0.801 |
| GFAP | 0.739 | 0.717 | 0.762 | 0.925 | 0.911 | 0.939 | 0.693 | 0.666 | 0.720 | 0.879 | 0.862 | 0.896 |
| p-Tau181 + aβ42/40+NfL+GFAP | 0.830 | 0.807 | 0.853 | 0.941 | 0.927 | 0.955 | 0.797 | 0.771 | 0.822 | 0.941 | 0.928 | 0.954 |
| MTx-%C | 0.821 | 0.805 | 0.837 | 0.986 | 0.982 | 0.991 | 0.597 | 0.577 | 0.617 | 0.916 | 0.904 | 0.928 |
| p-Tau181+ MTx-%C | 0.869 | 0.850 | 0.888 | 0.990 | 0.986 | 0.994 | 0.762 | 0.740 | 0.784 | 0.976 | 0.968 | 0.983 |
| aβ42/40+ MTx-%C | 0.884 | 0.870 | 0.899 | 0.980 | 0.972 | 0.988 | 0.784 | 0.761 | 0.807 | 0.954 | 0.942 | 0.967 |
| NfL+ MTx-%C | 0.840 | 0.825 | 0.856 | 0.995 | 0.993 | 0.997 | 0.608 | 0.586 | 0.631 | 0.921 | 0.907 | 0.934 |
| GFAP+ MTx-%C | 0.851 | 0.833 | 0.870 | 0.987 | 0.981 | 0.993 | 0.691 | 0.664 | 0.717 | 0.953 | 0.943 | 0.963 |
| p-Tau181+ MTx-%C + aβ42/40+NfL+GFAP | 0.888 | 0.872 | 0.905 | 0.970 | 0.960 | 0.981 | 0.787 | 0.762 | 0.813 | 0.981 | 0.971 | 0.991 |

**Table 3**: The statistical efficacy of models in predicting aβ+/-.

| Variable(s) | AUC  (95%CI) | sensitivity | specificity | PPV | NPV |
| --- | --- | --- | --- | --- | --- |
| p-Tau181 | 0.853  (0.806-0.900) | 0.754 | 0.844 | 0.791 | 0.813 |
| p-Tau181+ MTx-%C | 0.880  (0.837-0.923) | 0.816 | 0.805 | 0.750 | 0.861 |
| aβ42/40+ MTx-%C  (CU Aβ- and MCI Aβ+) | 0.891  (0.840−0.942) | 0.884 | 0.767 | 0.753 | 0.892 |
| p-Tau181+ MTx-%C  (CU Aβ- and AD dementia) | 0.997  (0.992−1) | 0.999 | 0.942 | 0.902 | 0.999 |
| aβ42/40+ MTx-%C  (MCI Aβ- and MCI Aβ+) | 0.788  (0.713-0.863) | 0.783 | 0.689 | 0.701 | 0.773 |
| p-Tau181+ MTx-%C  (MCI Aβ- and AD dementia) | 0.980  (0.961-1) | 0.935 | 0.946 | 0.915 | 0.959 |
| p-Tau181 / aβ42+ MTx-%C | 0.907  (0.869-0.945) | 0.860 | 0.831 | 0.784 | 0.893 |

NPV, negative predictive value; PPV, positive predictive value

**Table 4:** Interpretation of testing combining MemTrax and p-Tau181/ Aβ42 in two cut-off approach

| Low cutoff | High cutoff | Sensitivity | Specificity | Medium risk | PPV | NPV |
| --- | --- | --- | --- | --- | --- | --- |
| 0.200 | 0.270 | 0.909 | 0.752 | 0.055 | 0.730 | 0.918 |
| 0.170 | 0.280 | 0.917 | 0.752 | 0.088 | 0.741 | 0.922 |
| 0.180 | 0.280 | 0.909 | 0.755 | 0.077 | 0.741 | 0.915 |
| 0.190 | 0.280 | 0.909 | 0.760 | 0.066 | 0.741 | 0.917 |
| 0.200 | 0.280 | 0.909 | 0.762 | 0.062 | 0.741 | 0.918 |
| 0.170 | 0.290 | 0.917 | 0.752 | 0.091 | 0.739 | 0.922 |
| 0.180 | 0.290 | 0.908 | 0.755 | 0.080 | 0.739 | 0.915 |
| 0.190 | 0.290 | 0.908 | 0.760 | 0.069 | 0.739 | 0.917 |
| 0.200 | 0.290 | 0.908 | 0.762 | 0.066 | 0.739 | 0.918 |
| 0.170 | 0.300 | 0.916 | 0.763 | 0.102 | 0.748 | 0.922 |
| 0.180 | 0.300 | 0.907 | 0.766 | 0.091 | 0.748 | 0.915 |
| 0.190 | 0.300 | 0.907 | 0.771 | 0.080 | 0.748 | 0.917 |
| 0.200 | 0.300 | 0.907 | 0.772 | 0.077 | 0.748 | 0.918 |
| 0.160 | 0.310 | 0.925 | 0.756 | 0.135 | 0.754 | 0.925 |
| 0.170 | 0.310 | 0.916 | 0.768 | 0.106 | 0.754 | 0.922 |
| 0.180 | 0.310 | 0.907 | 0.771 | 0.095 | 0.754 | 0.915 |
| 0.190 | 0.310 | 0.907 | 0.776 | 0.084 | 0.754 | 0.917 |
| 0.200 | 0.310 | 0.907 | 0.778 | 0.080 | 0.754 | 0.918 |
| 0.150 | 0.320 | 0.925 | 0.762 | 0.168 | 0.772 | 0.921 |
| 0.160 | 0.320 | 0.925 | 0.773 | 0.146 | 0.772 | 0.925 |
| 0.170 | 0.320 | 0.916 | 0.785 | 0.117 | 0.772 | 0.922 |
| 0.180 | 0.320 | 0.907 | 0.788 | 0.106 | 0.772 | 0.915 |
| 0.190 | 0.320 | 0.907 | 0.793 | 0.095 | 0.772 | 0.917 |
| 0.200 | 0.320 | 0.907 | 0.794 | 0.091 | 0.772 | 0.918 |
| 0.150 | 0.330 | 0.925 | 0.775 | 0.175 | 0.784 | 0.921 |
| 0.160 | 0.330 | 0.925 | 0.786 | 0.153 | 0.784 | 0.925 |
| 0.170 | 0.330 | 0.916 | 0.797 | 0.124 | 0.784 | 0.922 |
| 0.180 | 0.330 | 0.907 | 0.800 | 0.113 | 0.784 | 0.915 |
| 0.190 | 0.330 | 0.907 | 0.804 | 0.102 | 0.784 | 0.917 |
| 0.200 | 0.330 | 0.907 | 0.806 | 0.099 | 0.784 | 0.918 |
| 0.150 | 0.340 | 0.923 | 0.775 | 0.182 | 0.780 | 0.921 |
| 0.160 | 0.340 | 0.923 | 0.786 | 0.161 | 0.780 | 0.925 |
| 0.170 | 0.340 | 0.914 | 0.797 | 0.131 | 0.780 | 0.922 |
| 0.180 | 0.340 | 0.906 | 0.800 | 0.120 | 0.780 | 0.915 |
| 0.190 | 0.340 | 0.906 | 0.804 | 0.109 | 0.780 | 0.917 |
| 0.200 | 0.340 | 0.906 | 0.806 | 0.106 | 0.780 | 0.918 |
| 0.150 | 0.350 | 0.921 | 0.782 | 0.197 | 0.782 | 0.921 |
| 0.160 | 0.350 | 0.921 | 0.792 | 0.175 | 0.782 | 0.925 |
| 0.170 | 0.350 | 0.912 | 0.803 | 0.146 | 0.782 | 0.922 |
| 0.180 | 0.350 | 0.903 | 0.806 | 0.135 | 0.782 | 0.915 |
| 0.190 | 0.350 | 0.903 | 0.810 | 0.124 | 0.782 | 0.917 |
| 0.200 | 0.350 | 0.903 | 0.812 | 0.120 | 0.782 | 0.918 |
| 0.160 | 0.360 | 0.921 | 0.798 | 0.179 | 0.788 | 0.925 |
| 0.170 | 0.360 | 0.912 | 0.809 | 0.150 | 0.788 | 0.922 |
| 0.180 | 0.360 | 0.903 | 0.812 | 0.139 | 0.788 | 0.915 |
| 0.190 | 0.360 | 0.903 | 0.816 | 0.128 | 0.788 | 0.917 |
| 0.200 | 0.360 | 0.903 | 0.818 | 0.124 | 0.788 | 0.918 |
| 0.160 | 0.370 | 0.920 | 0.798 | 0.182 | 0.786 | 0.925 |
| 0.170 | 0.370 | 0.911 | 0.809 | 0.153 | 0.786 | 0.922 |
| 0.180 | 0.370 | 0.902 | 0.812 | 0.142 | 0.786 | 0.915 |
| 0.190 | 0.370 | 0.902 | 0.816 | 0.131 | 0.786 | 0.917 |
| 0.200 | 0.370 | 0.902 | 0.818 | 0.128 | 0.786 | 0.918 |
| 0.160 | 0.380 | 0.919 | 0.805 | 0.190 | 0.791 | 0.925 |
| 0.170 | 0.380 | 0.910 | 0.815 | 0.161 | 0.791 | 0.922 |
| 0.180 | 0.380 | 0.901 | 0.818 | 0.150 | 0.791 | 0.915 |
| 0.190 | 0.380 | 0.901 | 0.822 | 0.139 | 0.791 | 0.917 |
| 0.200 | 0.380 | 0.901 | 0.824 | 0.135 | 0.791 | 0.918 |
| 0.160 | 0.390 | 0.918 | 0.811 | 0.197 | 0.796 | 0.925 |
| 0.170 | 0.390 | 0.909 | 0.822 | 0.168 | 0.796 | 0.922 |
| 0.170 | 0.400 | 0.908 | 0.822 | 0.172 | 0.795 | 0.922 |
| 0.170 | 0.410 | 0.907 | 0.828 | 0.179 | 0.800 | 0.922 |
| 0.170 | 0.420 | 0.907 | 0.835 | 0.182 | 0.807 | 0.922 |
| **0.170** | **0.430** | **0.907** | **0.841** | **0.186** | **0.815** | **0.922** |
| **0.170** | **0.440** | **0.907** | **0.841** | **0.186** | **0.815** | **0.922** |
| 0.170 | 0.450 | 0.905 | 0.841 | 0.193 | 0.811 | 0.922 |
| 0.200 | 0.270 | 0.909 | 0.752 | 0.055 | 0.730 | 0.918 |
| 0.170 | 0.280 | 0.917 | 0.752 | 0.088 | 0.741 | 0.922 |
| 0.180 | 0.280 | 0.909 | 0.755 | 0.077 | 0.741 | 0.915 |

NPV, negative predictive value; PPV, positive predictive value; n=275.


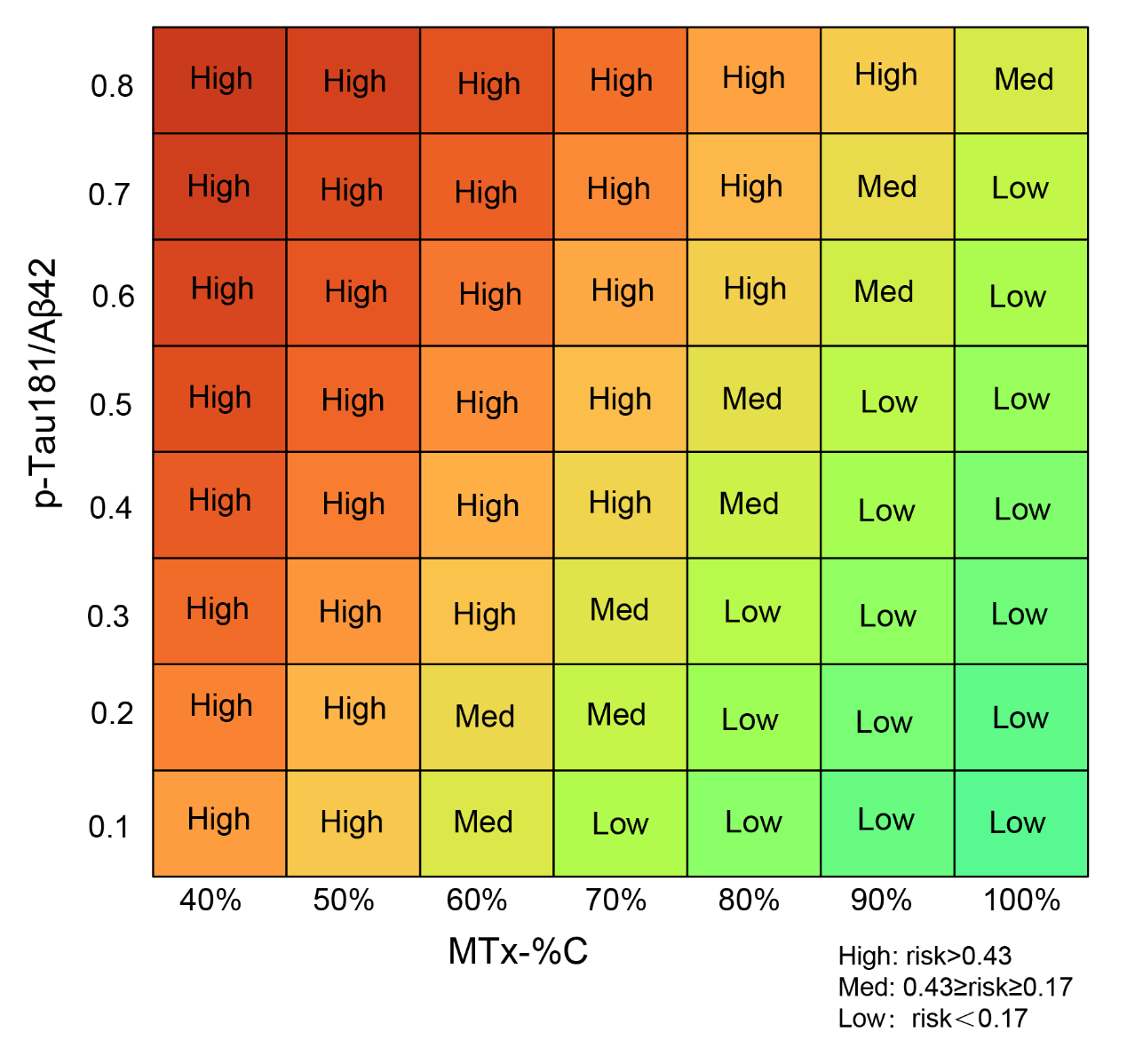


Figure 1: Heat map defines risk stratification thresholds for Aβ positive (High: risk>0.43, Medium: 0.43≥risk≥0.17, Low: risk＜0.17) derived from logistic regression models, using MTx-%C and p-Tau181/Aβ42 ratios.
